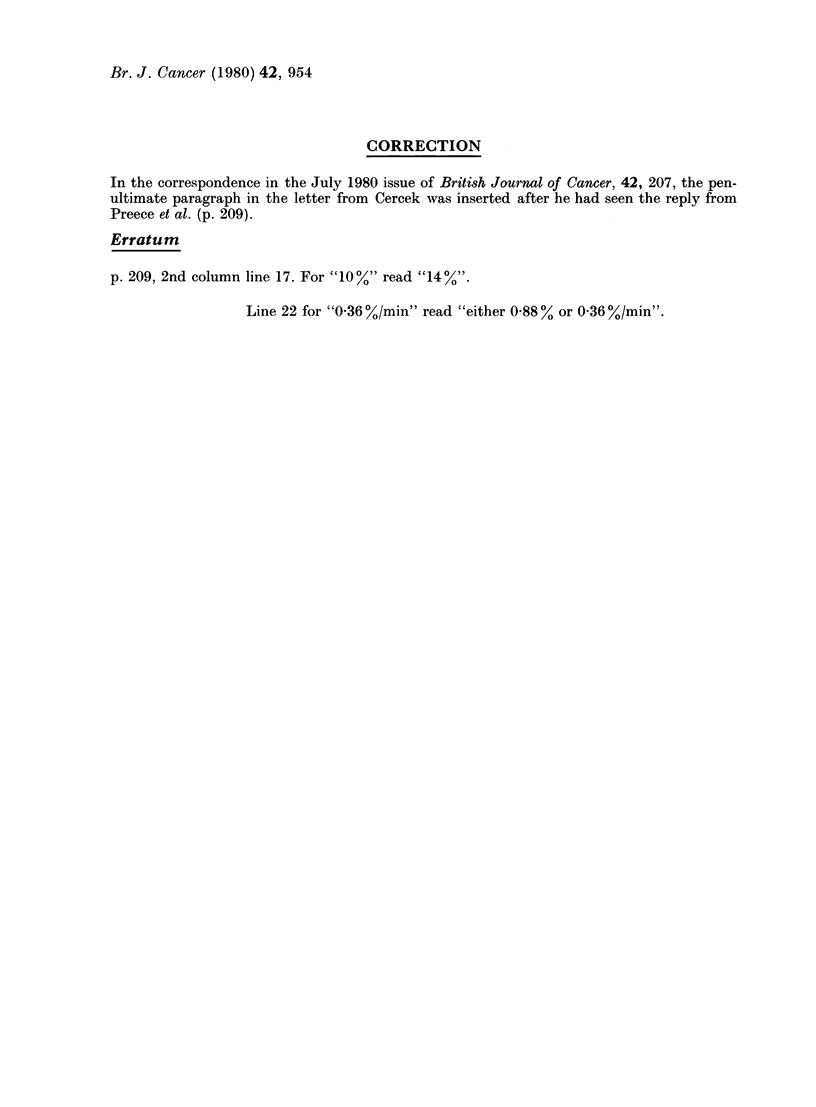# Correction

**Published:** 1980-12

**Authors:** 


*Br. J. Cancer* (1980) **42**, 954

### CORRECTION

In the correspondence in the July 1980 issue of *British Journal of Cancer*, **42,** 207, the penultimate paragraph in the letter from Cercek was inserted after he had seen the reply from Preece *et al.* (p. 209).

### *Erratum*

p. 209, 2nd column line 17. For "10%" read "14%".

Line 22 for "0·36%/min" read "either 0·88% or 0·36%/min".